# Standardized reporting of perioperative complications after male artificial urinary sphincter implantation

**DOI:** 10.1007/s00345-026-06246-y

**Published:** 2026-02-10

**Authors:** Navid Roessler, Malte W. Vetterlein, Tim H. J. Konrad, Robert J. Schulz, Max C. Wagner, Jakob Klemm, Shahrokh F. Shariat, Roland Dahlem, Margit Fisch, Tim A. Ludwig

**Affiliations:** 1 Department of Urology, University Medical Center Hamburg-Eppendorf, Martinistraße 52, 20246 Hamburg, Germany; 2 Department of Urology, Comprehensive Cancer Center, Austrian Comprehensive Cancer Center, Medical University of Vienna, Vienna, Austria; 3 Department of Urology, Weill Cornell Medical College, New York, NY USA; 4 Department of Urology, University of Texas Southwestern, Dallas, TX USA; 5 Department of Urology, Second Faculty of Medicine, Charles University, Prague, Czech Republic; 6 Hourani Center for Applied Scientific Research, Al-Ahliyya Amman University, Amman, Jordan; 7 Karl Landsteiner Institute of Urology and Andrology, Vienna, Austria

**Keywords:** Guideline adherence, Perioperative care, Postoperative complications, Quality of health care, Urinary sphincter, artificial

## Abstract

**Purpose:**

To systematically assess perioperative complications after artificial urinary sphincter (AUS) implantation using the European Association of Urology (EAU) complication reporting guidelines and evaluate long-term device outcomes.

**Methods:**

We retrospectively analyzed male patients undergoing AUS implantation between 2015 and 2020. Demographic, clinical, and surgical characteristics were described. Early complications within 6 weeks were captured using a procedure-specific complication catalog and graded according to the Clavien–Dindo Classification (CDC), in line with EAU standardized reporting recommendations. Primary endpoints were overall and major complications (CDC grade ≥ III); secondary endpoint was explantation-free survival.

**Results:**

A total of consecutive 227 AUS implantations (median age 73 years, IQR 67–76) were included. Severe stress urinary incontinence was predominantly post-radical prostatectomy (81%). Cuff types included distal double cuff (58%), bulbar single cuff (26%), and transcorporal cuff (16%). Complications occurred in 47% of cases, most frequently bleeding events (61%), followed by genitourinary (12%) and infectious events (11%). Major complications requiring a reintervention occurred in 7.9% of cases. Median follow-up was 52 months (IQR 32–66), with 5-year explantation-free survival of 73%. Explantation was required in 23% of cases, primarily due to urethral erosion and infection.

**Conclusion:**

AUS implantation is associated with frequent early complications, though most are minor. Applying a predefined, procedure-specific complication catalog according to EAU guidelines ensures systematic, comprehensive, and transparent reporting. This approach highlights clinically relevant events, supports cross-study comparability, and informs individualized patient counseling and perioperative management.

**Supplementary Information:**

The online version contains supplementary material available at 10.1007/s00345-026-06246-y.

## Introduction

Male stress urinary incontinence (SUI), most often following prostate surgery, remains a distressing and burdensome condition with major impact on quality of life [1–3]. Conservative measures are frequently attempted but have limited efficacy in moderate to severe cases, leaving surgery as the only durable option. Artificial urinary sphincter (AUS) implantation has been the gold-standard treatment since its introduction in 1974 [4]. Despite only minor modifications over the decades, the device continues to provide reliable long-term continence and high patient satisfaction, even in complex cases and after revision surgery [5–8]. Yet, these benefits must be balanced against procedure-related morbidity. AUS implantation carries a spectrum of peri- and postoperative complications that can range from minor and self-limiting to major adverse events necessitating revision or explantation [9, 10].

Accurate characterization of these risks is particularly relevant given the profile of contemporary AUS candidates: predominantly men with a history of prostate cancer treatment, advanced age, and multiple comorbidities. Because AUS implantation is an elective procedure, transparent and standardized morbidity assessment is critical for both patient selection and counselling. However, complications are often underreported, especially when minor, and definitions vary widely across studies, which makes unbiased, reproducible, and comparable assessments cumbersome [11].

To address this challenge, the European Association of Urology (EAU) has established Guidelines on Reporting and Grading of Complications after Urologic Surgical Procedures [12]. Evidence from other procedures shows that applying such structured frameworks not only improves the accuracy of complication reporting but also provides deeper insights when predefined complication catalogues are applied systematically to uniform patient cohorts [13–16]. To date, however, these criteria have never been applied to AUS implantation.

The present study aimed to fill this gap by systematically assessing early complications after AUS implantation at a tertiary referral center. Using a predefined complication catalog, we report early morbidity within 6 weeks postoperatively according to the EAU quality criteria, and we complement these findings with long-term device outcomes.

## Patients and methods

### Study population

This observational study included all male patients who underwent implantation of an AUS (AMS 800™, American Medical Systems, Minnetonka, MN, USA) at our institution between January 2015 and October 2020. Perioperative and follow-up morbidity data were prospectively collected from electronic medical records (Soarian Clinicals) and retrospectively analyzed. The study protocol was approved by the Institutional Review Board (2021-100628-BO-ff) and conducted in accordance with institutional standards. Eligible patients presented with moderate-to-severe SUI confirmed by preoperative urodynamic assessment. Patients with bladder capacity < 300 mL or detrusor overactivity were not considered candidates for AUS implantation and were excluded from the analysis.

### Definition, extraction, and grading of complications

Prior to data extraction, our working group predefined a comprehensive complication catalog encompassing potential adverse events following AUS implantation. For each event, explicit definitions were established based on the Common Terminology Criteria for Adverse Events (CTCAE) v6.0 (Supplemental Table 1). Complications were subsequently extracted from digitalized patient charts. Data collection and grading followed a structured protocol: all complications occurring within the first 6 weeks post-implantation [17]—the period until patients returned for device activation—were recorded and graded according to the validated and adapted Clavien-Dindo Classification (CDC) [18, 19]. Postoperative complications were classified into bleeding, genitourinary, infectious, gastrointestinal, cardiovascular, neurological, and wound subgroups.

### Surgical procedure and perioperative standard of care

All patients received perioperative intravenous antibiotic prophylaxis (cefuroxime and gentamicin) according to the institutional standard protocol. AUS implantation was performed following a standardized surgical protocol, as previously described [20]. Patients without urethral risk factors received a proximal bulbar single cuff, whereas those with risk factors—such as prior urethroplasty, pelvic radiotherapy, previous incontinence surgery, or preoperative evidence of urethral damage assessed by cystoscopy or urethrography—were treated with a distal double cuff. In cases of prior double cuff explantation or pronounced urethral fragility, a transcorporal cuff was implanted as a salvage option [21]. The AUS system remained deactivated postoperatively, and a 12 F Foley catheter was placed and left in situ for 3 days.

### Clinical and surgical characteristics

Preoperative data included patient demographics (age, body mass index), etiology of SUI, 60-min pad test results [22], comorbidities (coronary artery disease, hypertension, diabetes mellitus), history of pelvic radiotherapy, thromboembolic events, ongoing antithrombotic therapy, and previous urethral or incontinence surgeries. Intraoperative data comprised the American Society of Anesthesiologists (ASA) physical status, operative time, implanted AUS cuff type, and cuff size. Follow-up followed institutional protocols, with all patients returning 6 weeks post-implantation for device activation and clinical evaluation. Additional follow-ups were scheduled at 6 and 24 months, and every 2 years thereafter, supplemented by telephone contacts conducted by trained medical staff to retrospectively collect follow-up data. Follow-up was defined as the interval from AUS implantation to the last contact, either in person, by telephone, or at the time of device explantation.

### Statistical analyses

Descriptive analyses were performed to characterize the study cohort in terms of demographic, clinical, and surgical variables. In line with the EAU guidelines for standardized reporting (Table 1), two primary morbidity endpoints were defined: (1) overall complication rate within 6 weeks postoperatively and (2) rate of major complications, defined as CDC grade ≥ III. A secondary endpoint was explantation-free survival, defined as the interval from AUS implantation to explantation of the entire system or any component. Median follow-up time was estimated using the reverse Kaplan–Meier method, with deaths and device downsizing censored in the survival analysis. Statistical analyses were conducted using R (R Foundation for Statistical Computing, Vienna, Austria) and Stata (StataCorp. 2015, Stata Statistical Software: Release 14; StataCorp LP, College Station, TX, USA).

**Table 1:** Characterization of the European Association of Urology quality criteria for accurate and comprehensive reporting of surgical outcome and their implementation in 227 artificial urinary sphincter cases between January 2015 and October 2020

	EAU quality criteria	Implementation
1	Define the method of accruing data	Retrospective chart review and data extraction of digitalized charts (Soarian Clinicals)
2	Define who collected the data	A resident and a medical student, who was not involved in the patients’ treatment course, collected data
3	Indicate the duration of follow-up	Median follow-up was defined as the period from AUS implantation to the last in-person contact
4	Include outpatient information	Outpatient information was included
5	Include mortality data and causes of death	Mortality and causes of death were reported
6	Include definitions of complications	A predefined complication catalog including definitions was generated
7	Define procedure-specific complications	
8	Report intra- and postoperative complications separately	Intra- and postoperative complications were considered separately
9	Use a severity grading system for postoperative complications (avoiding the distinction minor/major)	The Clavien–Dindo classification was used
10	Postoperative complications should be presented in a table either by grade or by complication type (specific grades should always be provided; grouping is not accepted)	A detailed table of postoperative complications, including grading, was provided
11	Include risk factors	Well-known risk factors were included into the analysis
12	Include readmissions and causes	Patients were routinely reviewed 6 weeks post-implantation for device activation, with further follow-ups scheduled at 6 and 24 months and every 2 years thereafter; unplanned visits due to complications were also included in the follow-up
13	Include reoperations, types, and causes	Reoperations, types, and causes were mentioned
14	Include the percentage of patients lost to follow-up	Due to the retrospective design, all patients were followed at least until postoperative device activation

*AUS* artificial urinary sphincter, *EAU* European Association of Urology

## Results

### Descriptive analysis of clinical and surgical characteristics

The study population comprised 227 AUS implantation cases with a median age at surgery of 72 years (IQR 67–76). Most patients presented with severe SUI, reflected by a median 60-min pad test voiding volume of 100 mL (n = 171, IQR 54–106). The primary cause of SUI was prior radical prostatectomy (80%), followed by endoscopic treatment for benign prostatic obstruction (transurethral resection of the prostate: 7.5%; laser enucleation of the prostate: 2.2%). Reconstructive procedures accounted for 8.8% of cases (12 excisions with primary reanastomosis, five buccal mucosa graft urethroplasties, and one YV plasty). Other causes were rare (1.3%), including two ileal neobladders and one case of SUI after low-dose-rate brachytherapy without prior surgery. Comorbidities included hypertension (64%), coronary artery disease (26%), and diabetes mellitus (19%). Pelvic radiotherapy was reported in 44% of patients, comprising external beam radiotherapy in 40% and brachytherapy in 3.5%. Before AUS implantation, 25% of patients had undergone endoscopic treatment and 10% urethroplasty for stricture disease. Previous incontinence procedures included prior AUS implantation in 33% and male sling surgery in 9.7%. Baseline patient characteristics are summarized in Table 2. Preoperative physical status was classified as ASA ≤ II in 63% and ASA ≥ III in 37% of patients. Regarding cuff type, 58 patients (26%) received a bulbar single cuff, 132 (58%) a distal double cuff, and 37 (16%) a transcorporal cuff. Median operative time was 53 min (IQR 48–60), and the median cuff size was 4.5 cm (range 4–6).

**Table 2:** Clinical characteristics of 227 cases who underwent artificial urinary sphincter implantation for stress urinary incontinence between January 2015 and October 2020

Age (years), median (IQR)	72 (67–76)
Body mass index (kg/m^2^), median (IQR)	28 (25–30)
Stress urinary incontinence etiology, n (%)	
Radical prostatectomy	182 (80)
Transurethral resection of the prostate	17 (7.5)
Laser enucleation of the prostate	5 (2.2)
Post-reconstructive surgery	20 (8.8)
Other	3 (1.3)
Urine loss at 60 min pad test (mL), median (IQR); n = 171	100 (54–106)
Comorbidities, n (%)	
Hypertension	146 (64)
Coronary artery disease	58 (26)
Diabetes mellitus	44 (19)
Prior pelvic radiation therapy, n (%)	
External beam radiation therapy	91 (40)
Brachytherapy	8 (3.5)
History of thromboembolic events, n (%)	
Stroke	13 (5.7)
Transient ischemic attack	4 (1.8)
Other	4 (1.8)
Antithrombotic therapy, n (%)	
Acetylsalicylic acid	61 (27)
Direct oral anticoagulants	16 (7)
Coumarins	6 (2.6)
Prior urethral stricture surgery, n (%)	
Endoscopic management	56 (25)
Urethroplasty	23 (10)
Prior incontinence surgery, n (%)	
Artificial urinary sphincter	75 (33)
Retrourethral male sling	22 (9.7)

Percentages may not add up to 100%, as they are rounded

*IQR* interquartile range

### Assessment of perioperative 6-week complications

**Fig. 1:**
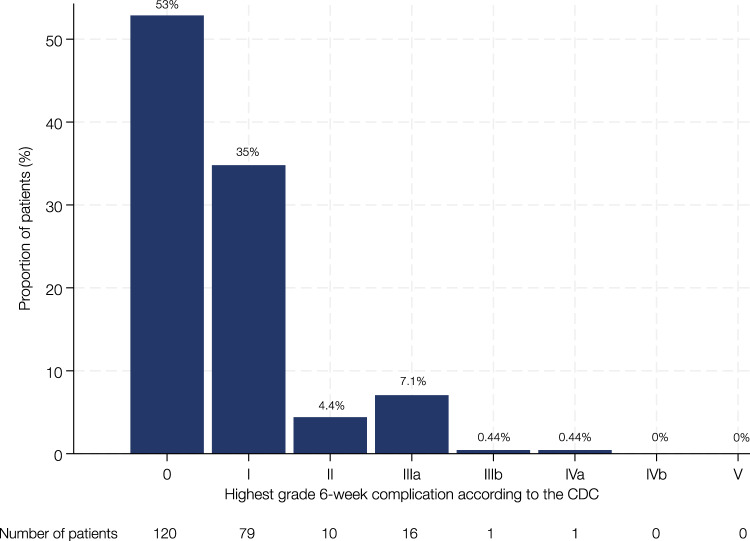
Column chart depicting the distribution of the highest grade 6-weeks complication per patient stratified by Clavien–Dindo classification

A detailed summary of the number, proportion, and grading of all complications according to the CDC is presented in Table 3. In total, 155 complications were recorded in 107 cases (47%, 95% confidence interval [CI] 41–54), corresponding to 120 patients (53%) without any reported complication during the first 6 weeks. Bleeding complications were most frequent (95 events, 61%), followed by genitourinary (18 events, 12%) and infectious complications (17 events, 11%). Other complications were rare (≤ 7%), including gastrointestinal, cardiovascular, neurological, and wound-related events. Overall, 18 major complications (CDC grade ≥ III) requiring intervention were identified among the 227 cases (7.9%, 95% CI 4.8–12). The distribution of complications by CDC grade is illustrated in Fig. 1.

**Table 3:** Frequencies, proportions, therapeutic management, and grading of postoperative 6-weeks complications in 227 artificial urinary sphincter implantations between January 2015 and October 2020

	CDC grading	Management	Number of complications	Proportion (n = 227) (%)
Bleeding^a^ 95 complications (61%) in 86 implantations				
Hematoma	I	Conservative; clinical observation or diagnostic evaluation only	79	35
Postoperative bleeding	I	Conservative; clinical observation or diagnostic evaluation only	15	6.6
Anemia	I	Conservative; clinical observation or diagnostic evaluation only	1	0.4
Genitourinary^a^ 18 complications (12%) in 17 implantations				
Transurethral catheter for urinary retention	IIIa	Transurethral catheter under radiological control	7	3.1
Suprapubic catheter for urinary retention	IIIa	Suprapubic catheter under local anesthesia	9	4
Acute kidney injury	I	Conservative; clinical observation or diagnostic evaluation only	2	0.9
Infectious^a^ 17 complications (11%) in 14 implantations				
Bacteriuria	II	Antibiotic treatment	11	4.8
Abscess	II	Conservative; clinical observation and antibiotic treatment	3	1.3
Epididymitis	II	Clinical observation, analgesic administration, and antibiotic treatment	2	0.9
Fever of unknown origin	II	Conservative; antipyretics, antibiotic treatment	1	0.4
Gastrointestinal^a^ 9 complications (5.8%) in 8 implantations				
Diarrhoea	I	Conservative; antidiarrhoeals, i.v. fluid support, electrolytes	4	1.8
Emesis	I	Conservative; antiemetics and i.v. fluid support	4	1.8
Enteric injury	IIIb	Appendiceal erosion caused by the AUS system, necessitating AUS explantation and appendectomy	1	0.4
Cardiovascular^a^ 8 complications (5.2%) in 7 implantations				
Hypertensive crisis	I	Clinical observation and antihypertensives	7	3.1
Angina pectoris	I	Conservative; clinical observation or diagnostic evaluation only	1	0.4
Neurological^a^ 7 complications (4.5%) in 6 implantations				
Dizziness	I	Conservative; clinical observation or diagnostic evaluation only	5	2.2
Stroke	IVa	Conservative; ICU	1	0.4
Syncope	I	Conservative; clinical observation or diagnostic evaluation only	1	0.4
Wound^a^ 1 complication (0.6%) in 1 implantation				
Wound infection	II	Conservative; clinical observation and antibiotic treatment	1	0.4

Percentages may not add up to 100%, as they are rounded

*AUS* artificial urinary sphincter, *CDC* Clavien–Dindo classification, *ICU* intensive care unit, *i.v.* intravenous

^a^The percentage refers to the proportion of all 155 complications

### Explantation-free survival

**Fig. 2:**
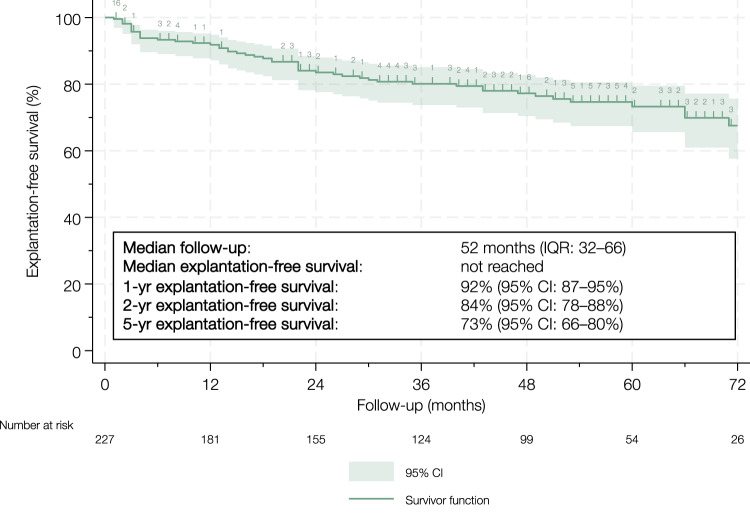
Kaplan–Meier curve illustrating explantation-free survival in 227 cases of artificial urinary sphincter implantation between 2015 and 2020. The curve is truncated at 72 months to minimize potential inaccuracies arising from the small number of patients remaining at risk beyond this time point

Median follow-up was 52 months (IQR 32–66). Estimated explantation-free survival was 84% (95% CI 78–88) at 2 years and 73% (95% CI 66–80) at 5 years (Fig. 2). Explantation was required in 52 cases (23%). The most common indication was urethral erosion (n = 20), followed by urethral erosion with infection (n = 14), infection alone (n = 6), urethral erosion with mechanical failure (n = 1), mechanical failure alone (n = 1), and mechanical failure with infection (n = 1). The remaining nine explantations included seven due to loss of function (one involving AUS downsizing) and two due to urethral stricture formation at the cuff site. During follow-up, one patient died from causes unrelated to AUS implantation.

## Discussion

We assessed 6-week postoperative complications after AUS implantation using a predefined complication catalog based on the EAU quality criteria for complication reporting, representing the first systematic application of these guidelines to AUS. Complications were frequent but mostly minor, with major events occurring primarily due to urinary retention. Kaplan–Meier analyses showed that approximately one in five patients required explantation over a median follow-up of 4.5 years, most commonly for urethral erosion. These findings underscore the importance of considering both early morbidity and long-term device-related risks in patient counseling and clinical decision-making.

Our analysis identified a broad spectrum of postoperative complications, with bleeding events being the most common, predominantly mild subcutaneous hematomas managed conservatively. Despite careful perioperative management of anticoagulants, bleeding risk remains relevant, particularly in elderly and multimorbid patients [23, 24]. Hematomas exemplify the value of standardized complication reporting: their clinical impact may range from negligible to clinically significant, with larger collections potentially impairing wound healing or predisposing to device explantation [24]. According to the CDC, any conservatively managed hematoma qualifies as a grade I complication. Consequently, even minor events contribute to overall morbidity rates. Studies that omit predefined, standardized tools risk underreporting such events, thereby underestimating morbidity and hampering cross-study comparability. Notably, postoperative bleeding has been identified as a risk factor for device explantation [24], illustrating that even seemingly minor or clinically “irrelevant” complications may carry prognostic significance.

Another example is asymptomatic bacteriuria. In the context of AUS implantation, this condition should be considered a complication, especially when antimicrobial treatment is initiated. Non-sterile urine in the early postoperative period may increase the risk of infection. In our cohort, bacteriuria occurred in nearly 5% of patients, corresponding to a CDC grade II complication, as antibiotic therapy is classified as grade II under strict CDC definitions.

Major complications in our series—defined as adverse events requiring reintervention—occurred predominantly due to urinary retention, which is usually manageable. Similar to prior studies, overall postoperative complication rates within 6 weeks after primary AUS implantation are around 35%, with urinary retention being the most common (31%) [17]. Nonetheless, careful management under radiologic control is essential, as repeated catheterization in fragile urethral tissue may predispose to cuff damage and erosion [17, 25]. Overall, our findings confirm that AUS implantation is a safe procedure: more than half of patients experienced no complications within the first 6 weeks postoperatively, even when applying a strict complication reporting framework as outlined by the EAU. This reinforces the position of AUS as the standard of care for men with moderate-to-severe SUI.

Reported AUS explantation rates range from 10 to 30% over follow-up periods of up to 10 years [9, 26, 27], though direct comparisons are limited by heterogeneous reporting practices. In a pooled analysis the overall reintervention rate was approximately 26% (range 15–45%), with a mean of 1.5 surgical procedures per patient [28]. Our relatively high rate of distal double and transcorporal cuffs reflects the large proportion of patients with urethral risk factors, such as prior radiotherapy or reconstructive surgery—both established predictors of erosion and device failure [29–32]. Furthermore, the predominance of older prostate cancer patients with multiple comorbidities highlights that contemporary AUS candidates represent a high-risk group. More than one-third were classified as ASA III–IV. Despite technical and perioperative advances, procedure-related morbidity remains considerable in this population [33].

Given the elective nature of AUS implantation and the potential impact of early complications on long-term outcomes, standardized morbidity reporting is crucial. Hematomas and bacteriuria exemplify how events often considered minor can influence overall complication rates and, in some cases, predispose to explantation. Without predefined and validated frameworks, such events are easily overlooked, resulting in inconsistent morbidity estimates and potentially misleading perceptions of risk. Combining standardized reporting with comprehensive perioperative assessment enhances transparency, allows meaningful cross-study comparison, and supports individualized, patient-centered counseling.

The interpretation of our findings is limited by the retrospective, single-center, and descriptive study design. As a reconstructive referral center, our cohort includes a high proportion of complex patients, including those with prior radiotherapy, prior urethral stricture treatment, or previous AUS implantation. Institutional practice at our center recommends the use of tandem cuffs in patients with fragile urethras [29], which contributed to the high tandem cuff rate observed. These factors likely increase complication rates compared with a standard first-time single-cuff population and may limit the generalizability of our findings to broader clinical settings. The 6-week window was chosen to ensure complete assessment of early complications and coincided with routine follow-up for device activation [17]. The small number of major adverse events precluded multivariable risk factor analysis, and the number of explantations limited statistical correlation between complications and device loss. Future multicenter studies with larger cohorts should examine early morbidity across different stages of AUS management—for example, whether patients undergoing revision or third-time implantation face different risks than treatment-naïve individuals. Moreover, evaluating the relationship between perioperative morbidity and patient-reported outcomes would provide valuable insight into the clinical relevance of both minor and major events, thereby refining perioperative management and counseling.

In summary, AUS implantation is associated with frequent but predominantly minor and manageable complications, while major complications are uncommon. Applying the EAU quality criteria enabled systematic, comprehensive, and transparent reporting, ensuring accurate identification of adverse events that might otherwise be overlooked, such as hematomas or bacteriuria. Even minor complications may influence patient experience and long-term device outcomes. Future studies should adopt standardized reporting frameworks and investigate the impact of perioperative morbidity on patient-reported outcomes, ultimately supporting evidence-based optimization of perioperative care.

## Supplementary Information

Below is the link to the electronic supplementary material.

Supplementary file1 (DOCX 21 KB)

## Data Availability

This observational study included all male patients who underwent implantation of an AUS (AMS 800™, American Medical Systems, Minnetonka, MN, USA) at our institution between January 2015 and October 2020. Perioperative and follow-up morbidity data were prospectively collected from electronic medical records (Soarian Clinicals). The study protocol was approved by the Institutional Review Board (2021-100628-BO-ff) and conducted in accordance with institutional standards.
