# Supplementary material for: Standardized reporting of perioperative complications after male artificial urinary sphincter implantation
Source: World J Urol. 2026 Feb 10;44(1):156. doi: 10.1007/s00345-026-06246-y (PMC12891164; doi:10.1007/s00345-026-06246-y)
Supplement: Supplementary file 1 — Supplementary file1 (DOCX 21 KB) [file 345_2026_6246_MOESM1_ESM.docx]

**Supplemental Table 1 –** Predefined complication catalog of general and procedure-specific perioperative complications, employing the Common Terminology Criteria for Adverse Events (CTCAE) v.6.0.

(<https://dctd.cancer.gov/research/ctep-trials/trial-development/ctcae-v6.0.xlsx>)

**Bleeding:**

1. **Anemia**: A disorder characterized by a reduction in the amount of hemoglobin in 100 mL of blood. Signs and symptoms of anemia may include pallor of the skin and mucous membranes, shortness of breath, palpitations of the heart, soft systolic murmurs, lethargy, and fatigability.
2. **Postoperative bleeding**: A disorder characterized by bleeding occurring after a surgical procedure.
3. **Hematoma**: A disorder characterized by a localized collection of blood, usually clotted, in an organ, space, or tissue, due to a break in the wall of a blood vessel.

**Genitourinary:**

1. **Acute kidney injury**: A disorder characterized by the acute loss of renal function (within 2 weeks).
2. **Urinary retention**: A disorder characterized by accumulation of urine within the bladder because of the inability to urinate.
3. **Urethral injury**: A finding of damage to the urinary system during a surgical procedure.

**Infectious:**

1. **Fever of unknown origin**: A disorder characterized by elevation of the body's temperature above the upper limit of normal without any explanation. Diagnosis of exclusion.
2. **Bacteriuria**: Asymptomatic bacteriuria with >10^5^ colony forming units/mL in urine culture. A disorder characterized by an infectious process involving the urinary tract, most commonly the bladder and the urethra.
3. **Abscess**: Collection of pus within the tissue of the body.
4. **Epididymitis**: Inflammation of the epididymis, typically caused by infection, resulting in pain and swelling in the scrotum. A disorder characterized by an infectious process involving the urinary tract, most commonly the bladder and the urethra.
5. **Pyelonephritis**: Symptomatic inflammation of the kidney without SIRS criteria, 10^5^ colony forming units/mL in urine culture. A disorder characterized by an infectious process involving the urinary tract, most commonly the bladder and the urethra
6. **Gastroenteritis**: Inflammation of the stomach or the small intestines, possibly involving diarrhea and vomiting. A disorder characterized by inflammation of the small and large intestines.

**Gastrointestinal:**

1. **Enteric injury**: Intraoperative finding of damage to the gastrointestinal system during a surgical procedure.
2. **Emesis**: A disorder characterized by the reflexive act of ejecting the contents of the stomach through the mouth.
3. **Diarrhoea**: A disorder characterized by an increase in frequency and/or loose or watery bowel movements.

**Cardiovascular:**

1. **Hypertensive crisis**: A disorder characterized by a pathological increase in blood pressure.
2. **Angina pectoris**: A disorder characterized by substernal discomfort due to insufficient myocardial oxygenation e.g., angina pectoris.

**Neurological:**

1. **Stroke**: A disorder characterized by an interruption of blood supply to parts of the brain resulting in neurological damage.
2. **Dizziness**: A disorder characterized by a disturbing sensation of lightheadedness, unsteadiness, giddiness, spinning or rocking.
3. **Syncope**: A disorder characterized by an episode of lightheadedness and dizziness which may precede an episode of syncope.

**Wound:**

1. **Wound infection**: A disorder characterized by an infectious process involving the wound.
2. **Wound dehiscence**: A finding of separation of the approximated margins of a surgical wound.
3. **Hernia**: Protrusion of abdominal contents through a weakness or defect in the abdominal wall. A finding of development of a new problem at the site of an existing wound.
4. **Reservoir herniation**: Displacement or protrusion of the implant reservoir into the inguinal or scrotal region through a weakness in the abdominal or inguinal wall. A finding of development of a new problem at the site of an existing wound.
